# The chemical states and atomic structure evolution of ultralow-energy high-dose Boron implanted Si(110) via laser annealing

**DOI:** 10.1038/s41598-017-13415-y

**Published:** 2017-10-12

**Authors:** Fu-Ying Lee, Zong-Zhe Wu, Li-Chi Kao, Feng-Mei Chang, Sheng-Wen Chen, Shiu-Ko JangJian, Hui-Yu Cheng, Wei-Liang Chen, Yu-Ming Chang, Kuang Yao Lo

**Affiliations:** 1 Department of Physics, National Cheng Kung University, Tainan, 701 Taiwan; 2 Taiwan Semiconductur Manufacturing Company, Tainan, Taiwan; 3 Center for Condensed Matter Sciences, National Taiwan University, Taipei, 106 Taiwan

## Abstract

Further scale down the dimension of silicon-based integrated circuit is a crucial trend in semiconductor fabrication. One of the most critical issues in the nano-device fabrication is to confirm the atomic structure evolution of the ultrathin shallow junction. In this report, UV Raman spectroscopy, X-ray photoelectron spectroscopy (XPS), X-ray absorption near edge structure (XANES) and reflective second harmonic generation (RSHG) are utilized to monitor the pulse laser induced atomic structure evolution of ultralow-energy high-dose Boron implanted Si(110) at room and cold substrate temperature. A peak feature around 480 cm^−1^ resolved in UV Raman spectra indicates the formation of Si-B bond after the laser irradiation. The red shift of binding energy of Si element (~99 eV) in XPS and the evolution of absorption peak (~196.2 eV) in XANES reveal that the changes in the chemical states of ultra shallow junction strongly correlate to the activation process of Boron implantation, which is confirmed by RSHG measurement. The substrate temperature effect in the recrystallization of Boron implanted region is also realized by cross-section high-resolution TEM (HRTEM). The phenomena of Si-B bond formation and ultra-shallow junction recrystallization can be traced and applied to improve the reliability of Si ultra shallow junction in the future.

## Introduction

A well reliable reproducibility and control of dopant purity, dosage and spatial distribution for the ultra-shallow doped layers can be achieved by ultralow-energy ion-implantation techniques and fast annealing process^1–3^. The main purpose of fast annealing process for the ultra-shallow junction (USJ) device in ULSI technology is to activate the dopant atoms and confine the dopant diffusion region by the fast annealing treatment, such as laser, spike and flash annealing^4–6^. Qualitative study for this near surface region of dopant-implanted Si after fast annealing process becomes an essential and important subject for semiconductor foundry to optimize the implant and annealing conditions as the nano-device scales down below 10 nm. Besides, the technology of cold implant was developed to enhance the amorphous thickness and to reduce the dislocation defect via annealing process^7–9^. The usage of cold substrate in ion implantation will reduce the damage in end-of-range (EOR) and suppress the occurrence of activation anomalies, dopant diffusion and leakage current^9–11^. However, it is hard to control the expected USJ since the structure evolution via fast annealing treatments are complicated and their control parameters are critical.

Particularly, it becomes a complicated problem to inspect the electrical properties and recrystallization degree of USJ below 10 nm length scale after annealing treatment. Due to the leakage current in the penetrated region beneath the USJ, Four-point probe will unavoidably penetrate into the USJ and obtain inaccurate sheet resistance^12^. Second ion mass spectroscopy (SIMS) only provides the depth analysis of dopant diffusion but no information about the recrystallization. The distribution analysis of SIMS would generate error signal nearby surface region which belongs the main depth of USJ^13^. More and further analyses tools with non-destruction should be developed for accurately inspecting the variation of defects and structure in the era of below 10 nm. However, the complex phenomena in the implanted region with ultrathin thickness bring about the difficulties on reliable analyses. Recently, ultraviolet (UV) Raman spectroscopy was performed on annealed USJ^6,14,15^. UV Raman spectroscopy reveals the variation between the amorphous phase and recrystallized phase. However, in this work we propose to utilize additional optical techniques to acquire more detail information about the chemical state and atomic structure evolution of the ultrathin implanted layer.

X-ray photoelectron spectroscopy (XPS) provides information about the chemical configuration in the depth of several nanometers. XPS is a useful tool to identify the composition of specimen and to determine the chemical configuration of the specific elements by the binding energy of valence electrons^16,17^. The electron configuration of Si-B bond can reveal the structural evolution of the complex amorphous layer at the surface (<10 nm) region of the implanted Si matrix. XPS provides more information about chemical configuration on the structure evolution of Si matrix through different laser annealing process^18^. Furthermore, X-ray absorption near edge structure (XANES) is sensitive to the unoccupied state of atoms^19,20^. The analysis of XANES is complex since the final state of the excited electron involves molecular orbital, crystal field, and multiple scattering, therefore, information about local symmetry, geometry of molecular, and oxidation state would lead to different features of XANES spectra^19^. Note that the probe depth of XANES is only the topmost few nanometers of specimen and close to the implantation depth of the semiconductor process. The formation of Si-B bond can be identified in the XPS and XANES spectra. Besides, reflected second harmonic generation (RSHG) method has been demonstrated to be a sensitive tool to explore the atomic structure information, such as the surface reconstruction, the dopant distribution and defect formations, of the implanted silicon after rapid thermal annealing (RTA)^12,21,22^.

Here we utilize the aforementioned optical methods to investigate the chemical states and atomic structure evolution of the low-energy and high-dose Boron implanted Si(110) wafer under different substrate temperature and post-annealing treatments. The structural transformation from amorphous toward crystalline and the variation of chemical composition are explored with these accessible techniques. The mutli-optical methods with non-destruction provide reliable inspection way in USJ within 10 nm and create a monitor window to study the science and technology of new generation semiconductor fabrication.

## Results and Discussion

### Sample description

In order to simply classify the samples in this work, B implanted Si(110) prepared at the substrate temperature of 25 °C and −60 °C were named as ***R*** sample and ***C*** sample, respectively. Samples annealed with constant pulse number (500 pulses) but varying the laser pulse energy and samples annealed with constant pulse energy (300 mJ) but varying the pulse number were named as a series of ***P*** and ***E*** processes, respectively.

### SIMS of ultralow-energy, high-dose B implanted Si(110) and cold Si(110)

**Figure 1:**
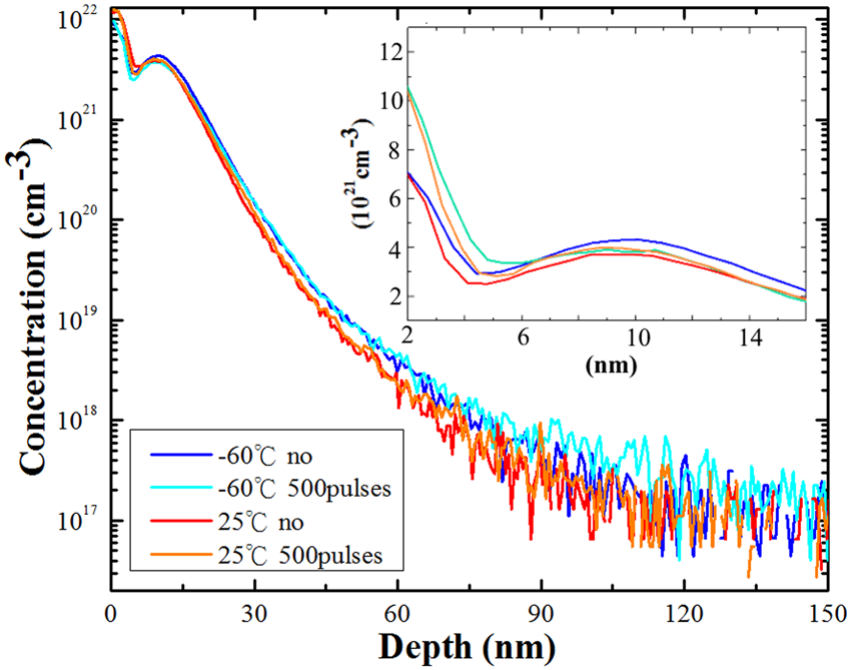
SIMS profiles for high-dose B implanted Si(110) at room temperature (25 °C) substrate and cold temperature (−60 °C) substrate, and their SIMS profiles after laser annealing with laser energy of 225 mJ and pulse number of 500. The insert is the close-up figure in linear scale (from 2 nm to 16 nm).

Figure 1 shows SIMS diagrams for ***R*** and ***C*** samples, and their SIMS profiles after laser annealing with laser energy of 225 mJ and pulse number of 500. These SIMS profiles are similar. Inspecting the close-up figure inserted in Fig. 1, B concentration at the depth of 5–10 nm in the C sample is higher than the one in the ***R*** sample. In the ***C*** sample, little B concentration increases in the depth of 2–7 nm after laser annealing. Besides, SIMS profiles after pulsed laser annealing shift toward the surface. In this work, KrF excimer laser (wavelength: 248 nm, and pulse duration: 15 ns) was used to perform non-melt laser annealing on ultrashallow junction. As the very high-heating and high-quenching rates of the pulsed laser anneal cause a large degree of supercooling of the amorphous Si phase, the B dopants are trapped in the lattice sites even high B concentrations^23^. Besides, on the basic of the short absorption length (5 nm at the wavelength of 248 nm), the fast electron-photon energy transfer (10^−11^ s) and higher diffusivity of B atom in ion implanted amorphous silicon^24,25^, the diffusion trend of B atoms is toward to the surface after annealing with short-pulsed KrF excimer laser. Contrary, long duration of rapid thermal annealing induces unexpected and wide distribution (see Supplementary Fig. S1). The treatment of laser annealing with short duration will limit the distribution of dopant and is suitable for next generation semiconductor fabrication.

### High-resolution TEM (HRTEM)

**Figure 2:**
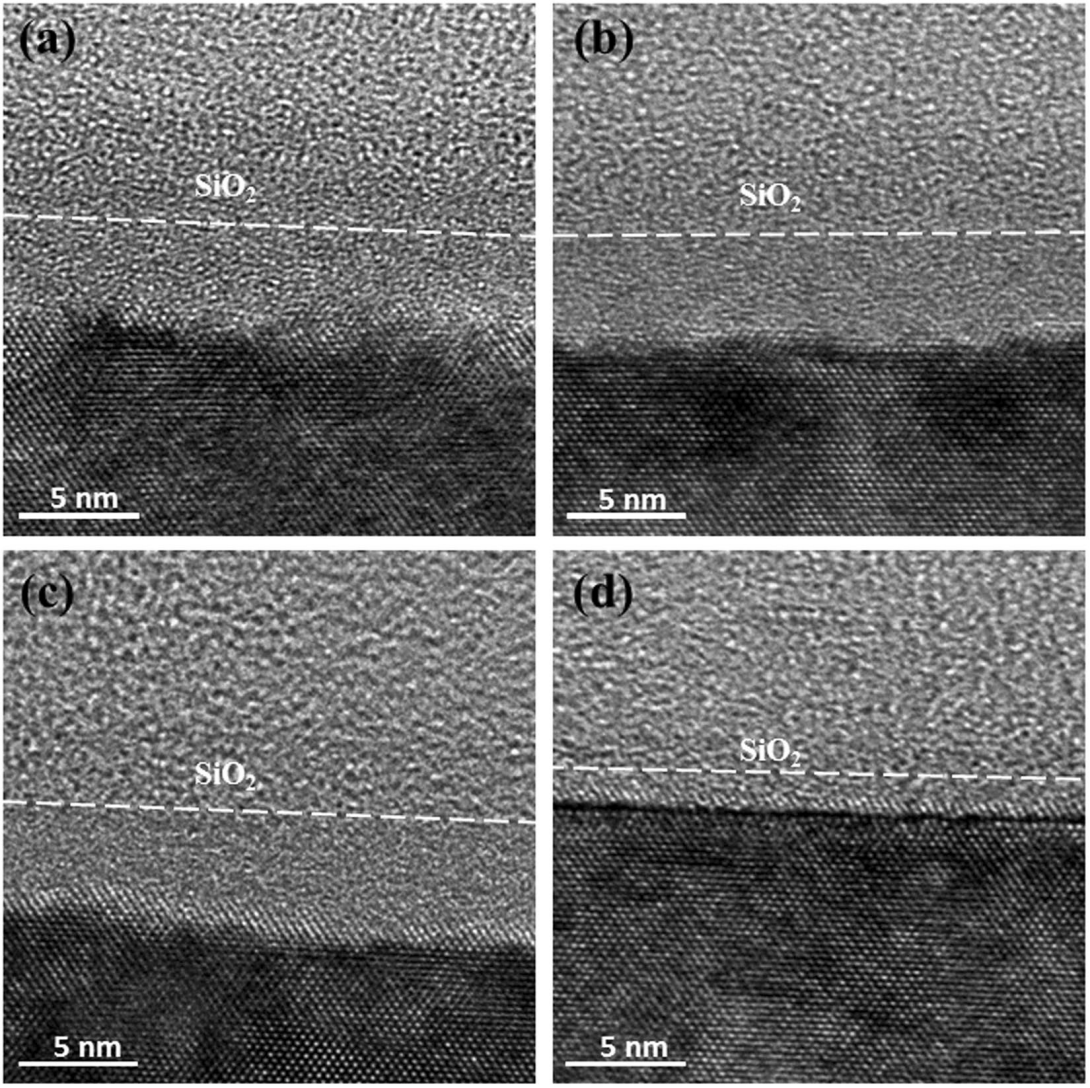
HRTEM images of high-dose B implanted Si(110) at (**a**) room temperature substrate and (**b**) cold temperature substrate. HRTEM images of high-dose B implanted Si(110) at (**c**) room temperature and (**d**) cold temperature with laser annealing of 300 mJ and 500 pulses. The oxide layer is located above the white dashed line.

Figure 2(a) and (b) show HRTEM images of ***R*** and ***C*** samples, respectively. Figure 2(c) and (d) show HRTEM images of ***R*** and ***C*** samples with laser annealing of 300 mJ and 500 pulses, respectively. All HRTEM images were taken at almost same diffraction condition, specimens were carefully tilted to <110> zone as close as possible. An oxide layer of around 5 nm thick is observed on the surfaces of every sample. The amorphous layers beneath oxide layers were formed by the bombardment of B ions during implantation, and were measured to be about (a) 4.4, (b) 4.7, (c) 3.9 and (d) 1.1 nm from their corresponding HRTEM images. These induced amorphous layers are almost same in thickness for samples at both implantation conditions. However, the interface of amorphous layer and crystalline Si of that implanted at low temperature is not as rough as that one implanted at room temperature, by comparing Fig. 2(a) and (b). The amorphous layer of low temperature implantation decreased significantly after pulse laser annealing, while the one at room temperature did only a little after the same annealing, Fig. 2(c) and (d).

Lattice points of Si in Fig. 2(b),(c) and (d) were nearly sharp through the whole image, but those in Fig. 2(a) showed different image characteristics, which have more blurred lattice points due to EOR defects. Defects in the under layer (silicon layer) in the ***R*** sample are higher than those in the ***C*** sample, and not completely removed after annealing, as shown in Fig. 2(c). The results agree with the relationship between the implanted substrate temperature and defects in EOR^9^. As a result, the above analyses indicate there is a better crystal structure for the ***C*** samples after laser annealing.

### UV Raman Spectroscopy

**Figure 3:**
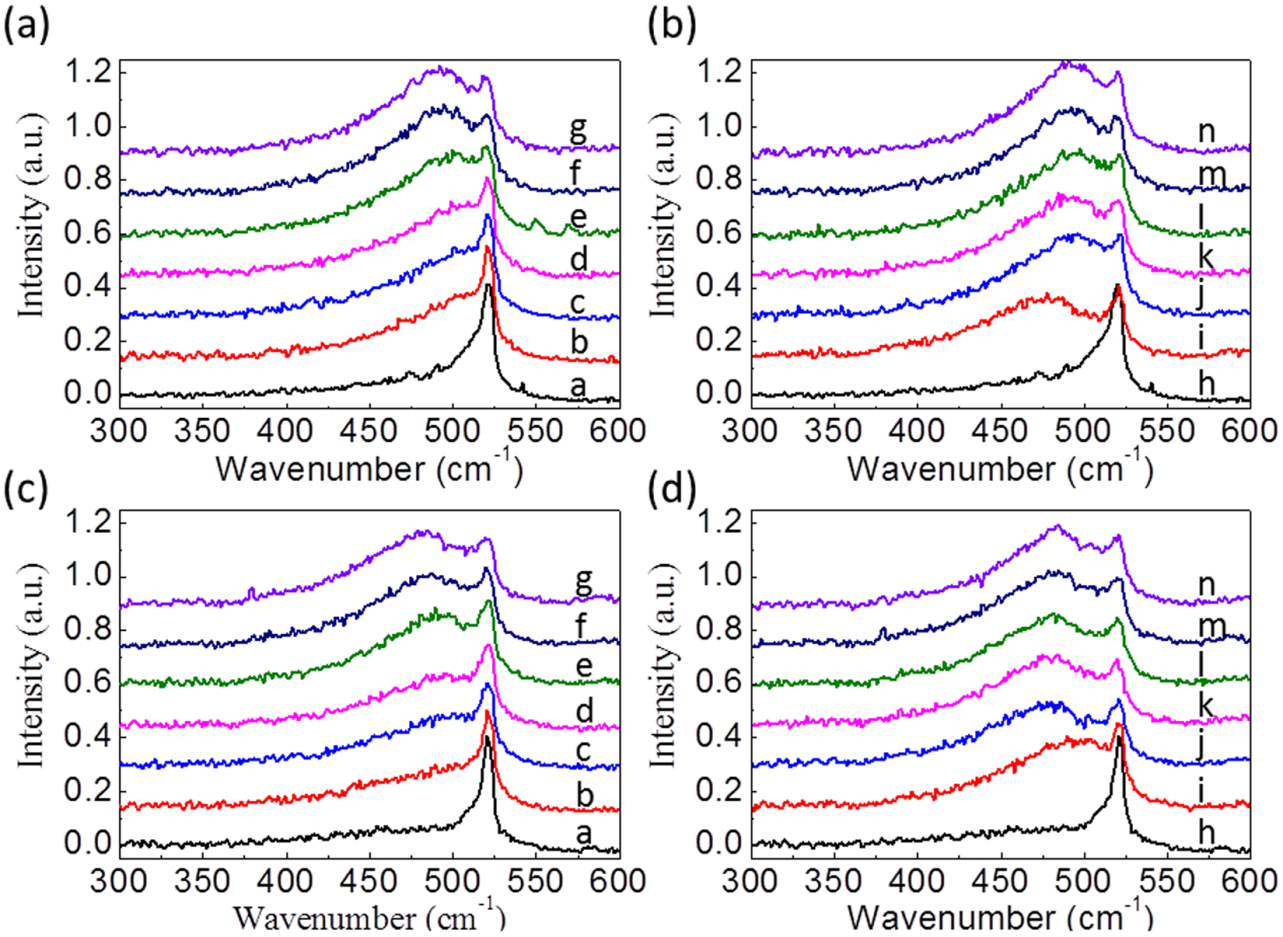
(**a**) Raman spectra for the ***R*** samples that were annealed in a series of ***P*** process. Labels (**a**–**g**) corresponds to “no annealing,” 175 mJ, 200 mJ, 225 mJ, 250 mJ, 275 mJ, and 300 mJ (**b**) Raman spectra for the ***R*** samples that were annealed in a series of ***E*** process. Labels h-n corresponds to “no annealing, 5, 50, 100, 250, 500, and 1000 pulses. (**c**) and (**d**) Show similar plots as in (**a**) and (**b**) but for the ***C*** samples.

**Figure 4:**
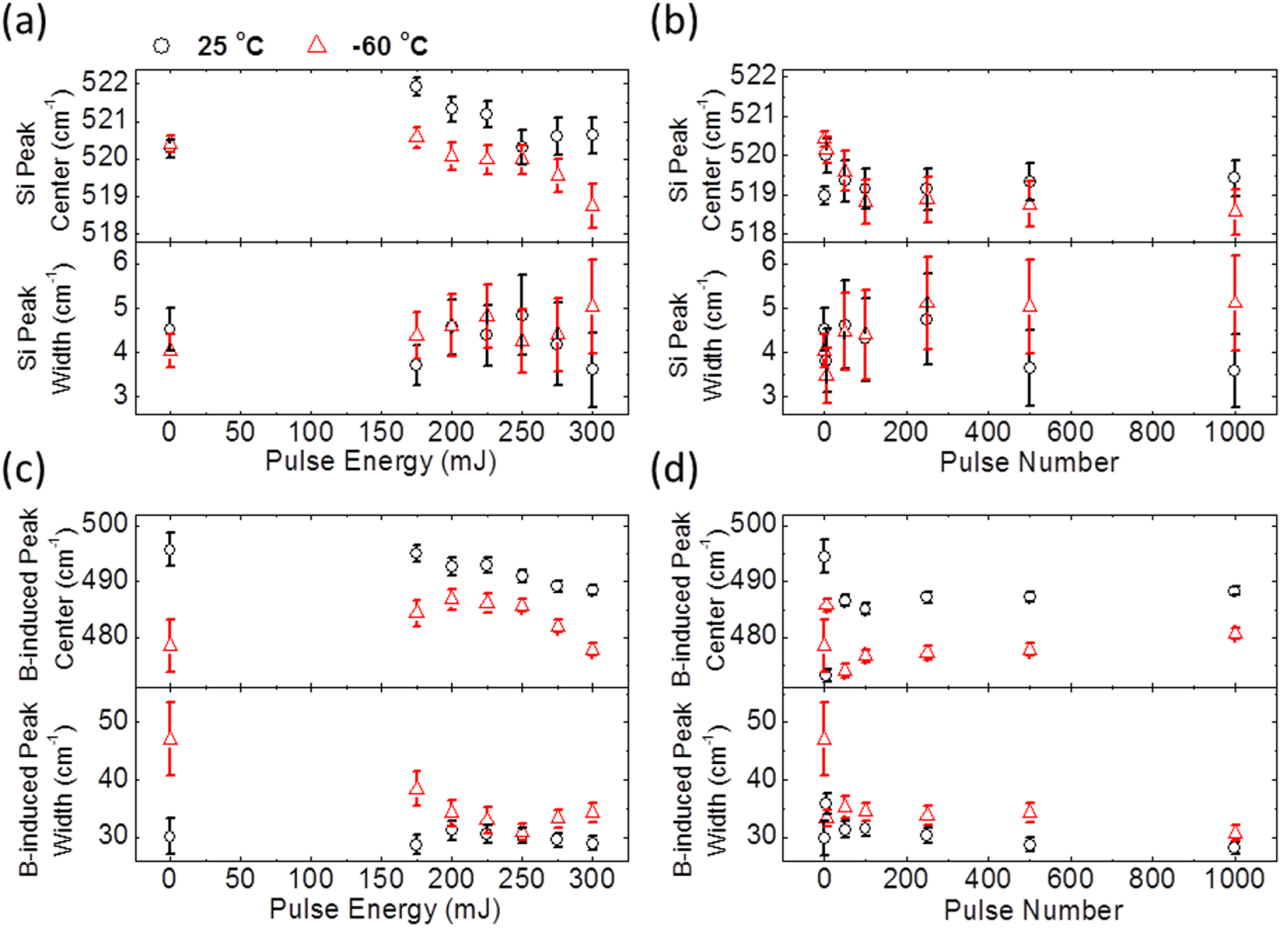
Peak center and peak width as determined by curve fitting. Si peak center and peak width for (**a**) a series of ***P*** processes, and (**b**) a series of ***E*** processes. B-induced peak center and peak width for (**c**) a series of ***P*** processes and (**d**) a series of ***E*** processes. Black circles and red triangle symbols are for ***R*** and ***C*** samples, respectively.

Figure 3(a) and (c) show the Raman spectra of ***R*** and ***C*** samples with a series of ***P*** process, and Fig. 3(b) and (d) show the Raman spectra of ***R*** and ***C*** samples with a series of ***E*** process, respectively. All the obtained Raman spectra were then curve-fitted by a sum of two Lorentzian peaks, with the center of the broad peak feature constrained from 470 to 515 cm^−1^, and the sharp silicon peak constrained between 518 and 522 cm^−1^. The detailed description of the curve-fitting procedure can be found in Supplementary Fig. S2. The fitting results are plotted in Fig. 4 to reveal the evolution of Si and B-induced phonon peaks, which can be correlated to the atomic structure in the ultra-shallow junction of Si surface during the laser annealing process. Figures 4(a) and 4(b) show the evolution of Si phonon mode as a function of the laser pulse energy or the laser pulse number, respectively. Meanwhile, the fitting results for the Boron-induced phonon mode are shown in Fig. 4(c) and (d). It is worth to emphasize that the conventional RTA annealing treatment of the same series of samples reveals no clear B-induced broad peak feature in UV Raman spectra as shown in Supplementary Fig. S3. This spectral discrepancy can be attributed to the well activation of Boron dopants in Si matrix via enough annealing time in RTA process. However, RTA process can’t confine the dopant distribution in ultra-shallow junction as indicated in Supplementary Fig. S1.

### XPS

**Figure 5:**
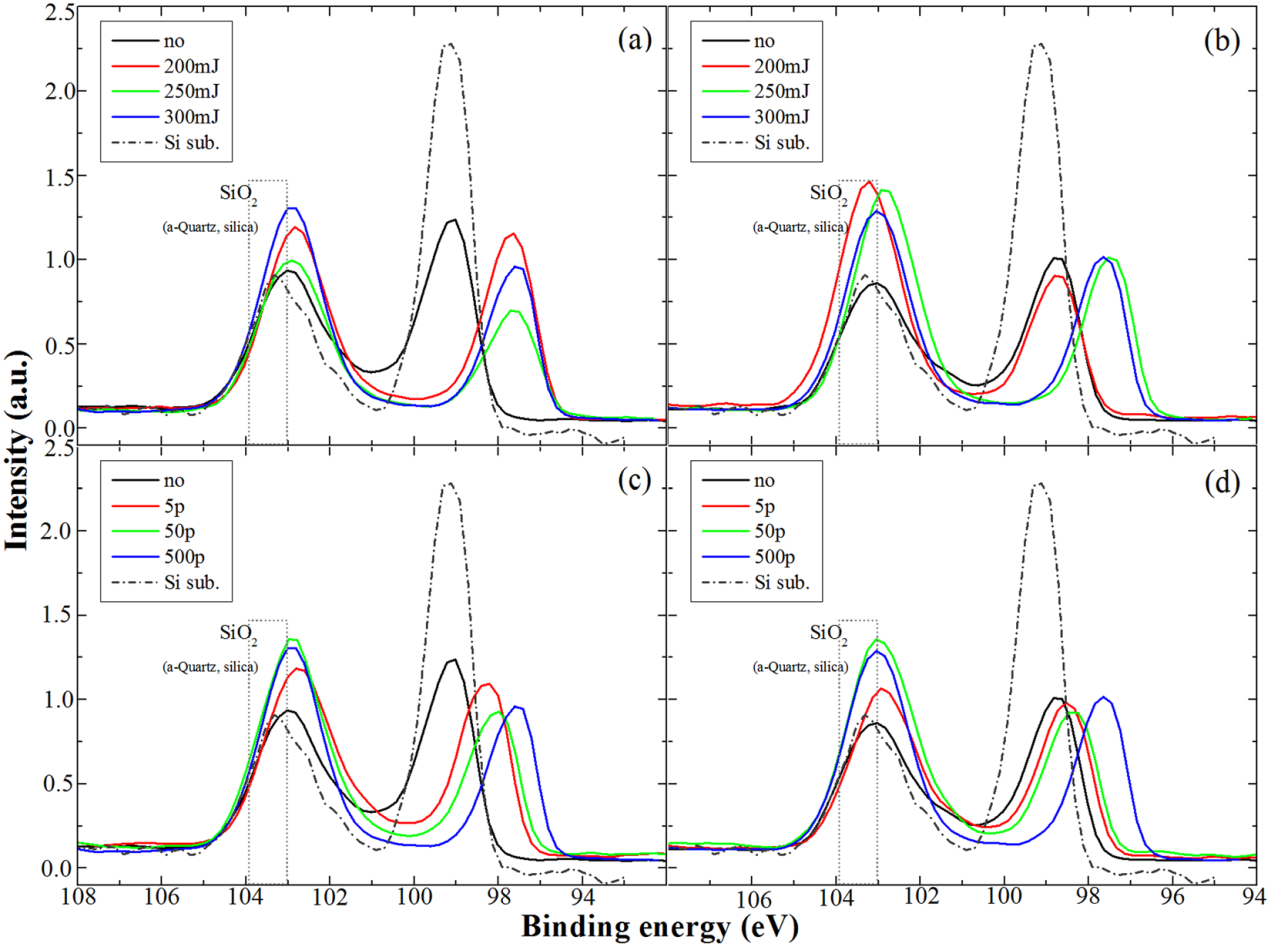
XPS spectra for (**a**) the ***R*** and (**b**) ***C*** samples in a series of ***P*** process. XPS spectra for (**c**) the ***R*** and (**d**) ***C*** samples in a series of ***E*** process.

From XPS spectra, shown Fig. 5, the binding energy of Si element (~99 eV) and amorphous type silica (~103 eV) have obvious shifts after laser annealing treatment for both implantation conditions. As excimer laser annealing treatment was not performed in vacuum, further oxidization is unavoidable. In a series of ***P*** process (shown in Fig. 5(a) and (b)), the peak of silicon element shifts toward to lower binding energy (97.6 eV) as the pulse energy increases. The shift of binding energy is caused by the formation of Si-B bond during laser annealing process. In the molecular orbital theory^26^, the energy of Si sigma (2 P) for formed Si-B bond is lower than the one for Si-Si bond. The obvious peak shift of Si element is due to the fact that B dopants with high concentration were halted on the surface layer (~5–10 nm), and further formed Si-B bond via short-duration laser annealing. Except the peak of binding energy of Si element shifts to 97.4 eV, variation in XPS after laser annealing depends on the substrate temperature.

Inspect the evolution of chemical states in the ***R*** and ***C*** samples with a series of ***E*** process (shown in Fig. 5(c) and (d)), only the irradiation of 5 pulses can provide enough thermal budge to exhibit obvious shift of Si 2p in the ***R*** sample. However, more irradiation shots (500 pulses) are necessary to supply enough thermal budge to activate B dopant in the ***C*** sample. There are similar behaviors for the ***R*** and ***C*** samples with a series of ***P*** processes, as shown in Fig. 5(a) and (b). The pulse energy of 200 mJ can provide enough thermal budge to form stable Si-B bond in the ***R*** samples, but more pulse energy (among 200 mJ and 250 mJ) is required to form Si-B bond in the ***C*** samples. As B ions bombard on Si(110) at 25 °C, larger thermal vibration of Si lattice will weaken the energy of injected B ions. The thickness of amorphous layer is shorter than the ones at −60 °C, as shown in Fig. 2(a) and (b). Less energy of laser irradiation can cause the formation of Si-B bond in the ***R*** samples. Laser irradiation with more energy is needed to activate B dopant in the amorphous region of the ***C*** samples, even though recrystalline process was faster in the ***C*** samples.

### XANES

**Figure 6:**
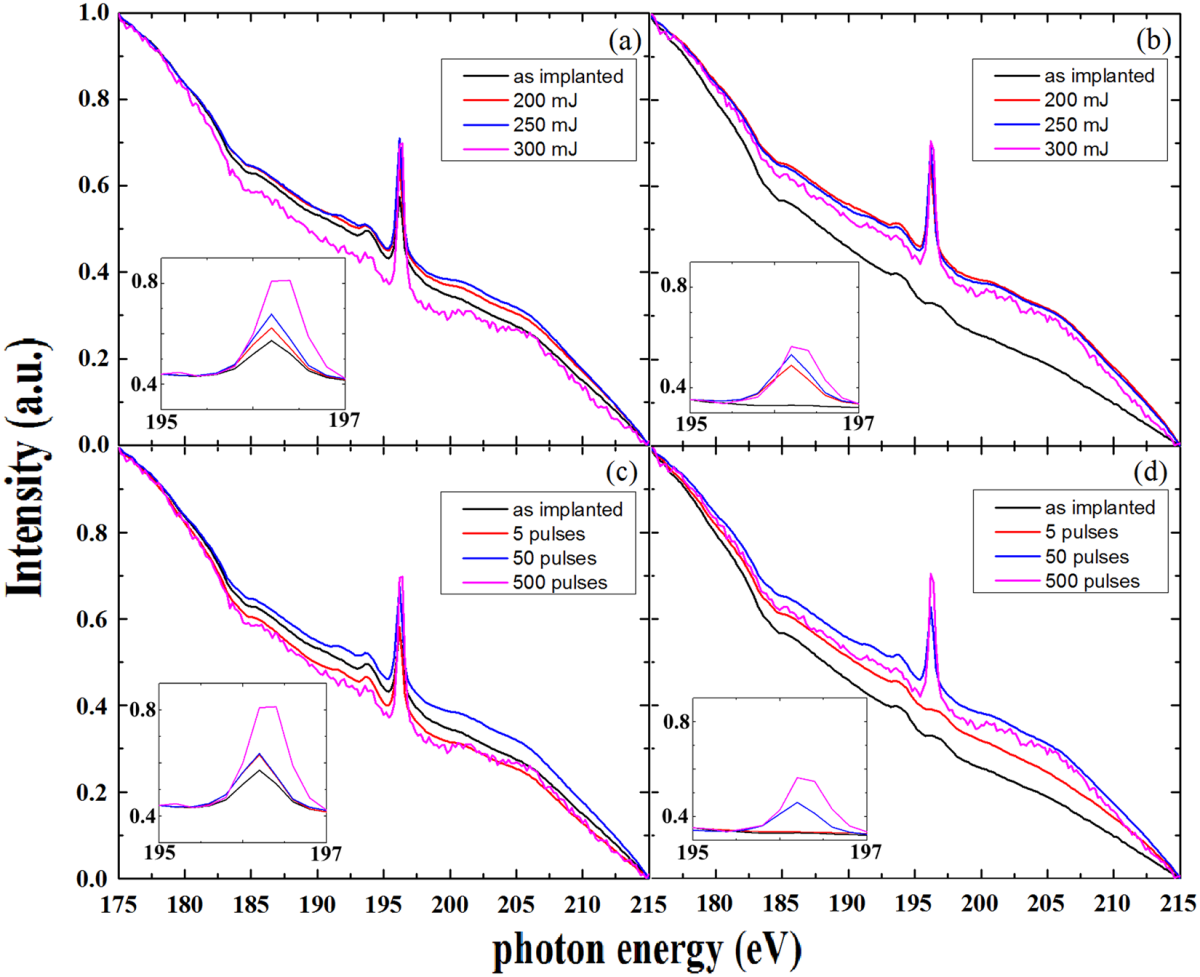
XANES spectra for (**a**) the ***R*** and (**b**) ***C*** samples in a series of ***P*** process. XANES spectra for the (**c**) the ***R*** and (**d**) ***C*** samples in a series of ***E*** process. The inserts are the close-up figures of normalized XANES spectra (from 192 eV to 197 eV).

In order to further confirm chemical states of B dopant after laser annealing, we perform XANES experiments with NSRRC synchrotron light source, which was carried out by total electron yield (TEY) mode^27^, as shown in Fig. 6. This kind of XANES measurement modes could reveal information about electron configuration near the surface. There is an absorption peak of 196.2 eV in the XANES spectra, which depends on the laser energy and implantation condition. We normalized the spectra and enlarged the characteristic region to observe the results clearly, as shown in the insert of Fig. 6. The absorption peak is mainly dominated by the 1 s→${\sigma }^{\ast }$ transition^28^. Based on molecular orbital theory, If the absorption peak belongs to ${{\rm{B}}}_{2}{{\rm{O}}}_{3}$, there is not only 1 s→${\sigma }^{\ast }$ but also exist the transition of 1 s→${\pi }^{\ast }$ about 194 eV, which did not exhibit in XANES spectra^29^. Therefore, the absorption peak at 196.2 eV in XANES would not come from the composition of boron oxide and crystalline of boron due to discrepancies on the electron configuration^29,30^. The peak at around 196.2 eV in XANES should contribute from Si-B bond and its amplitude increases as the energy or pulse number of pump laser increase. The results agree with analyses of XPS and UV Raman, which the form of Si-B bond would be enhanced by the thermal budge of laser irradiation.

The difference of the structure evolution between the ***R*** and ***C*** samples can be obtained from the discrepancies in XANES spectra. The peak intensity of around 196.2 eV increases as the pulse number or pulse energy increases at both series of ***P*** and ***E*** processes. The trend of XANES spectra is close to the changes in XPS spectra. Especially, the binding energy of Si-B bond shifts close to 98 eV in XPS (shown in Fig. 5), and then the peak of around 196.2 eV in XANES would appear. The peak of 196.2 eV exists in the ***R*** sample without laser annealing (shown in Fig. 6(a) and (c)), however, there is no such peak in the ***C*** sample without laser annealing or with no enough laser irradiation (shown in Fig. 6(a) and (c)). During implantation process, Si-B bond would be formed in the surface layer in the ***R*** sample due to self-annealing effect caused by large collision between lattice vibration of Si matrix and injected B ions. Besides, there is no shift of binding energy in XPS (shown in Fig. 5(b)) but an obvious peak of 196.2 eV in XANES spectrum in the ***C*** sample with the laser energy of 200 mJ in the ***P*** processes (shown in Fig. 6(b)). The resolution of XANES is less than 3 nm which is shorter than the one of XPS. The changes of Si-B bonds in the surface layer would be more obviously revealed in XANES spectra.

### RSHG

RSHG experiments were performed to confirm the polar structure evolution of B implanted Si(110) with room and cold temperature substrate via laser annealing treatment. The atomic structure of Si(110) surface breaks the centrosymmetric structure of Si bulk and exhibits 2 mm and 4 mm symmetrical dipole contribution^31^.

**Figure 7:**
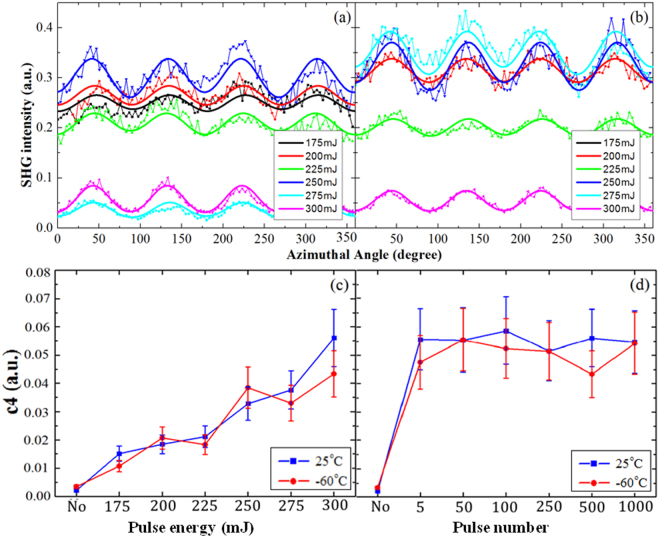
RSHG pattern for (**a**) the R samples and (**b**) C samples in a series of ***P*** process. The simulated *c*_4_ for the ***R*** and ***C*** samples a series of (**c**) ***P*** and (**d**) ***E*** processes.

In a series of ***P*** processes, the *s-p* RSHG (s-wave fundamental light and p-wave RSHG output light) patterns are similar 4 mm symmetry but their amplitude and bias are dependent on the irradiated pulse power, as shown in Fig. 7(a) and (b) for the ***R*** and ***C*** samples, respectively. The formation of polar Si-B bonds based on activation degree and reflects to the value of 4 mm symmetrical dipole, *c*_4_^21,22^. Figure 7(c) shows the correlation with the simulated *c*_4_ and irradiated laser energy for the ***R*** and ***C*** samples, which indicates the value of simulated *c*_4_ increases with the laser power. In a series of ***E*** processes, the correlation with the simulated *c*_4_ and irradiated laser pulses for the ***R*** and ***C*** samples is shown in Fig. 7(d). The values of *c*_4_ keep a constant for different irradiated pulse number. From Fig. 7(c) and (d), there is no obvious discrepancy between the ***R*** and ***C*** samples. The laser energy of 300 mJ per pulse is enough to activate B in the implanted Si even the pulse number is few. However, weaker laser energy can not activate B dopant in the implanted Si even more laser pulses. RSHG experiments exhibit a fact of that the activation of B dopant in Si matrix is strongly dependent on irradiated laser power.

The laser annealing treatment has less annealing duration (15 ns for each pulse) to limit the diffusion length of B dopant, indicated in SIMS diagram (Fig. 1). However, SHG patterns acquired from the ***R*** and ***C*** samples with RTA treatment and their simulated *c*_4_ are more precise, as shown in Supplementary Fig. S4, since the diffusion of B is obvious in SIMS for RTA treatment (shown in Supplementary Fig. S1).

## Discussion

The aforementioned experimental evidences exhibit that the recrystallization of Boron implanted Si(110) via laser annealing can lead to the formation of Si-B covalent bond in the topmost 10 nm region. In the UV Raman spectra, one can easily identify the formation of a broad phonon feature around 480 cm^−1^ in addition to the Si phonon mode around 520 cm^−1^. It is worth to note that the 480 cm^−1^ broad peak appears and its intensity increases as the pulse energy or pulse number increases as shown in Fig. 3. This result only appears in the case of pulse laser annealing but not in the case of conventional RTA treatment as shown in Fig. S3. In general, one will expect a mixture of Si-B composite, silica, amorphous Si and Si crystalline in the surface layer of Boron implanted Si(110) before and after the laser annealing. For the as-implanted Si sample, there is no broad peak feature in its UV Raman spectrum, but an obvious silica peak in its XPS. Based on these spectroscopic evidences one can exclude the origin of 480 cm^−1^ broad peak from silica or amorphous Si.

The broad-characteristic Si-B peak easily appeared at the case of room temperature substrate under low laser power, as shown in Fig. 5. The result is agreed with XPS because of that larger shift happened in the ***R*** samples via laser annealing with lower laser energy or less laser pulses. Besides XANES spectra have no Si-B peak in the ***C*** sample without laser annealing or with low laser energy. These optical analyses agree with the results of advantage of the cold temperature substrate. The usage of cold substrate will lead the implanted region to be more amorphous and suppress the tunneling of implanted ion, and mutli-optical analyzed results from comparison with the case of room temperature would reveal the detailed distinguish in structure evolution.

## Conclusion

All-optical techniques, including UV Raman spectroscopy, XPS, XANES and RSHG, were successfully utilized to characterize the chemical states and atomic structural evolution of ultralow-energy high-dose Boron implanted Si(110) under different substrate temperatures and laser annealing conditions. The experimental results exhibit the discrepancy of the atomic structure evolution between the two sample sets prepared at 25 °C and −60 °C, respectively. Short irradiated duration of laser pulses suppresses the diffusion of B dopant in Si matrix during the laser annealing process. According to UV Raman spectra, a broad feature at 480 cm^−1^ is assigned to the B-induced phonon mode and the appearance of this peak indicates the formation of B-Si covenant bond in the ultra-shallow junction of B-implanted Si(110). XPS and XANES techniques furthermore offer the detail chemical states analysis to support the conclusion derived from the UV Raman and RSHG measurements. In this work, we have demonstrated that all-optical techniques and their spectroscopic analyses can open a window to realize the actual atomic structure evolution in the ultra-shallow junction of B-implanted Si(110) during the laser annealing process, which can guide the design and fabrication of ultra-shallow junction in the topmost 10 nm of silicon surface.

## Methods

### Sample preparation

The implantation process of 12 inch Si(110) wafers was fabricated by Taiwan Semiconductor Manufacturing Company (TSMC)^32^. As Si(110) substrate has better hole mobility and The implantation process were treated with two substrate temperature, at room (25 °C) and cold temperature (−60 °C). These Si(110) samples were implanted with B ions with an energy of 1.0 keV and a dose of 5.0 × 10^15^ atoms/cm^2^.

Laser annealing process was performed in air by a KrF excimer laser with wavelength of 248 nm and pulse duration time of 15 ns. The energy of pulse laser can be tuned from 175 mJ to 300 mJ. A convex lens was set to achieve a focus area of 1 cm^2^. In this work, pulse power and irradiated shot are the parameters for varied annealing treatment.

### UV Raman measurement

The UV Raman spectroscopy was performed with a home-built confocal micro-Raman setup under ambient condition. A 325 nm, 35 mW, TEM_00_, and CW He-Cd laser (KIMMON: IK3301R-G) was used as the excitation light source. It was focused onto the sample with a 40X UV objective (OFR, LMU-40X-NUV**)**. The laser irradiation power was estimated to be ~7.5 mW on sample surface. The backscattering Raman signal was collected by the same objective and re-focused into an optical fiber, which relayed the optical signal into the high-resolution spectrometer (Horiba Jobin-Yvon, FHR-640) integrated with a liquid nitrogen cooled CCD detector (Horiba Jobin-Yvon, Symphony). Since a 2400 gvs/mm grating blazed at 400 nm was chosen and the entrance slit width was set to 50 μm, the ultimate spectral resolution of the spectrometer achieved ~0.8 cm^−1^ in the obtained UV Raman spectra.

### XPS experiments

XPS measurement was carried out by PHI Versa Probe III with the incident photon energy 2 KeV and energy resolution 0.1 eV. Then we fitted the data with Gaussian to compare the difference between our laser annealing samples.

### XANES experiments

We do the XANES experiments at TLS beamline 20A1 (continuous X-ray light source) in NSRRC, Taiwan. The incident photon energy could be tuned from 60 eV to 1250 eV by changing the gratings with four different groove density. The beam size of X-ray focused at sample is about 1.5 mm × 1.0 mm.

### RSHG experiments

RSHG experiment setup was detailed in ref.^22^. The laser source is a pulsed 20 Hz Q-switched Nd-YAG laser with a wavelength of 1064 nm, and a pulse duration time of 6 ns. The power of laser spot was limited to 50 mW/cm^2^ to prevent the annealing effect and the damage to the sample. The azimuthal dependence of the sp-RSHG intensity was measured by the p-wave polarized RSHG with the irradiation of s-wave polarized fundamental light as the sample rotated stepwise.

## Electronic supplementary material

Supplementary information
