# Supplementary material for: The chemical states and atomic structure evolution of ultralow-energy high-dose Boron implanted Si(110) via laser annealing
Source: Sci Rep. 2017 Oct 12;7:13022. doi: 10.1038/s41598-017-13415-y (PMC5638925; doi:10.1038/s41598-017-13415-y)
Supplement: Supplementary file 1 — Supplementary information [file 41598_2017_13415_MOESM1_ESM.pdf]

The chemical states and atomic structure evolution of ultralow-energy high-dose Boron implanted Si(110) via laser annealing

Fu-Ying Lee[1], Zong-Zhe Wu[1,#], Li-Chi Kao[1], Feng-Mei Chang[1], S-W Chen[2], Shiu-Ko JangJian[2], Hui-Yu Cheng[3], Wei-Liang Chen[3], Yu-Ming Chang[3,*] and Kuang Yao Lo[1,*]

1. Department of Physics, National Cheng Kung University, Tainan 701, Taiwan
2. Taiwan semiconductor manufacturing company, Tainan, Taiwan
3. Center for Condensed Matter Sciences, National Taiwan University, Taipei 106, Taiwan

RTA experimental process and experimental results

The RTA process of our samples was performed with MILA-3000 Rapid Thermal Annealer in a 5 torr N_2 gas environment. The RTA temperatures of these samples were set at T = 1100 ^{o}C with 25 ^{o}C increment.

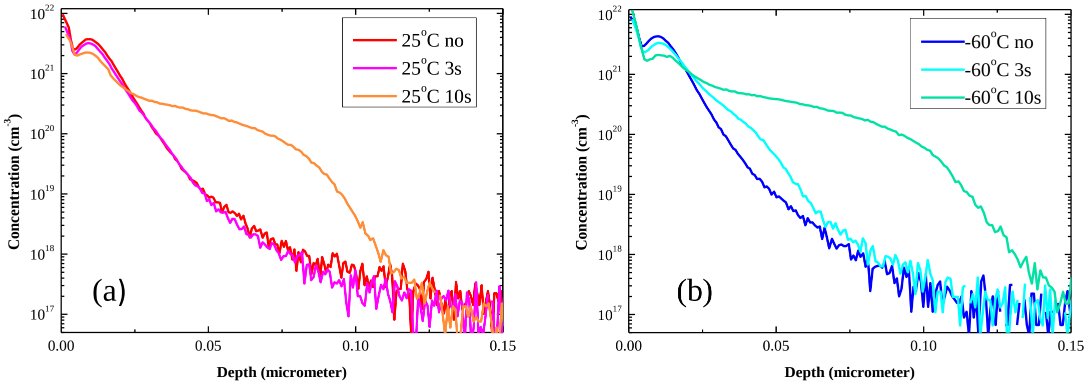

Figure S1 SIMS diagram for B implanted Si(110) with (a) room and (b) cold temperature substrate via RTA treatment

Curve fitting of UV Raman Spectrum: an example

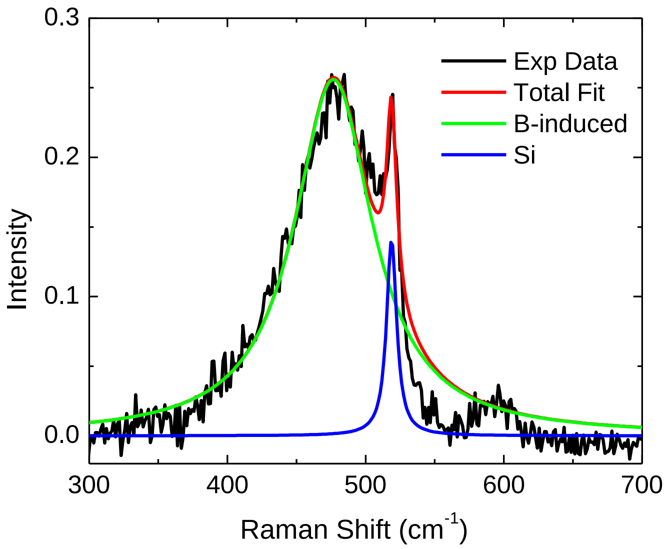

Figure S2: Curve fitting for the -60 ^{o}C doping sample annealed with 100 pulses of 300 mJ pulse energy. Black line is the experiment data, and red line is the curve fitting result. Green and red lines are respectively the profiles of fitted B-induced and Si peaks.

UV Raman spectra of Boron-implanted Si(110) under RTA treatment

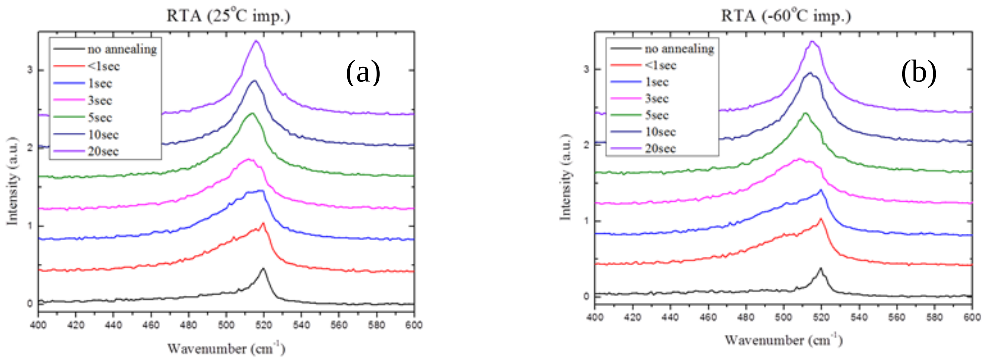

Figure S3: UV Raman spectra of B-implanted Si(110) samples via RTA treatment. The initial B-implantation was performed at (a) 25 ^{o}C and (b) -60 ^{o}C substrate temperature, respectively.

RHG on implanted Si(110) with RTA process

Through RTA treatment, B dopants diffuse widely, shown in Fig.S1, and amorphous layer was recrystallized via longer duration of thermal treatment. RSHG

patterns for both series implanted Si(110) with RTA process were shown in Fig. S4(a) and (b), and the trend of simulated c_4 were indicated in Fig. S4(c)

It is clear to inspect the trend of c_4 with RTA time. c_4 value increases with RTA times as the implanted region was gradually recrystallized and activated. Si-B bonds were built with the symmetry of Si(110) surface, and then c_4 value decreases as B dopants diffuse out of this region. It is hard to distinguish the dopant behavior and recrystallization from the case of room and cold substrate temperature.

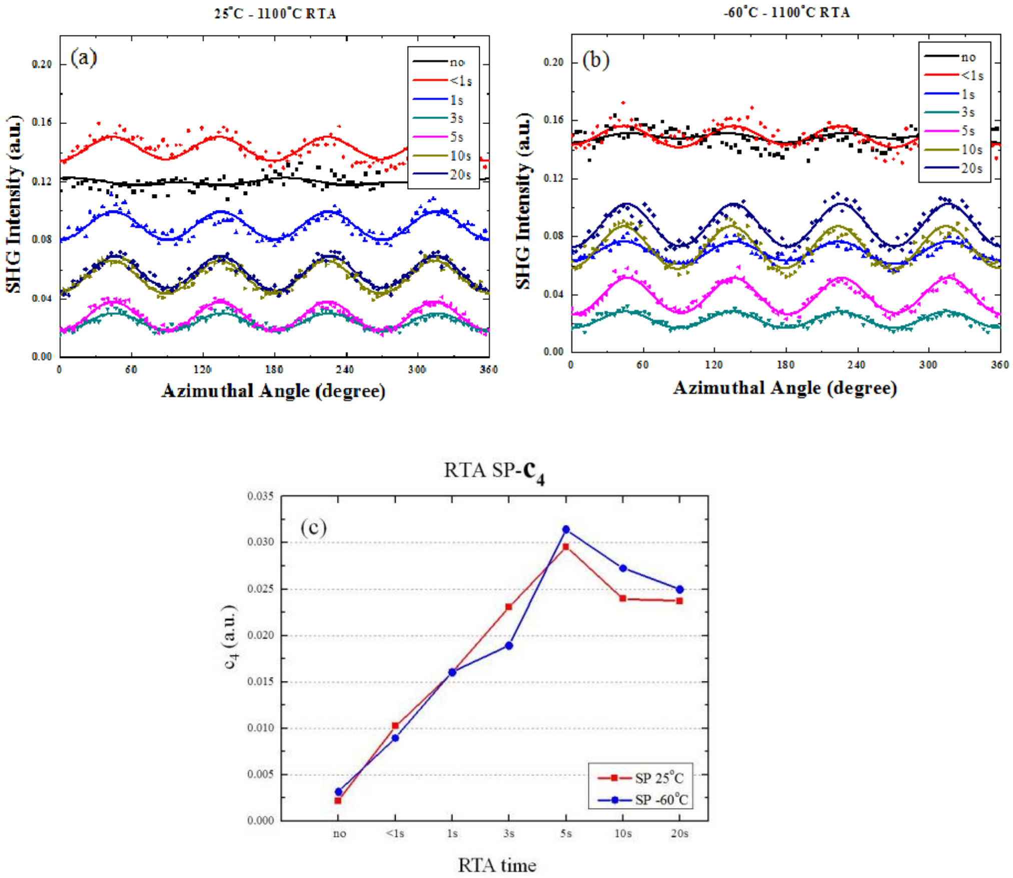

Figure S4 RSHG diagram for B implanted Si(110) with (a) room and (b) cold substrate via RTA treatment. (c) the simulated value of c_4 from RSHG pattern of (a) and (b)
